# Predictors of severity and mortality among patients hospitalized with COVID-19 in Rhode Island

**DOI:** 10.1371/journal.pone.0252411

**Published:** 2021-06-18

**Authors:** Aakriti Pandita, Fizza S. Gillani, Yiyun Shi, Anna Hardesty, Meghan McCarthy, Jad Aridi, Dimitrios Farmakiotis, Silvia S. Chiang, Curt G. Beckwith

**Affiliations:** 1 Department of Medicine, University of Colorado School of Medicine, Denver, Colorado, United States of America; 2 Division of Infectious Diseases, Warren Alpert Medical School of Brown University, Providence, Rhode Island, United States of America; 3 Department of Internal Medicine, Warren Alpert Medical School of Brown University, Providence, Rhode Island, United States of America; 4 Department of Pediatrics, Division of Pediatric Infectious Diseases, Warren Alpert Medical School of Brown University, Providence, Rhode Island, United States of America; SUNY Downstate: SUNY Downstate Health Sciences University, UNITED STATES

## Abstract

**Background:**

In order for healthcare systems to prepare for future waves of COVID-19, an in-depth understanding of clinical predictors is essential for efficient triage of hospitalized patients.

**Methods:**

We performed a retrospective cohort study of 259 patients admitted to our hospitals in Rhode Island to examine differences in baseline characteristics (demographics and comorbidities) as well as presenting symptoms, signs, labs, and imaging findings that predicted disease progression and in-hospital mortality.

**Results:**

Patients with severe COVID-19 were more likely to be older (p = 0.02), Black (47.2% vs. 32.0%, p = 0.04), admitted from a nursing facility (33.0% vs. 17.9%, p = 0.006), have diabetes (53.9% vs. 30.4%, p<0.001), or have COPD (15.4% vs. 6.6%, p = 0.02). In multivariate regression, Black race (adjusted odds ratio [aOR] 2.0, 95% confidence interval [CI]: 1.1–3.9) and diabetes (aOR 2.2, 95%CI: 1.3–3.9) were independent predictors of severe disease, while older age (aOR 1.04, 95% CI: 1.01–1.07), admission from a nursing facility (aOR 2.7, 95% CI 1.1–6.7), and hematological co-morbidities predicted mortality (aOR 3.4, 95% CI 1.1–10.0). In the first 24 hours, respiratory symptoms (aOR 7.0, 95% CI: 1.4–34.1), hypoxia (aOR 19.9, 95% CI: 2.6–152.5), and hypotension (aOR 2.7, 95% CI) predicted progression to severe disease, while tachypnea (aOR 8.7, 95% CI: 1.1–71.7) and hypotension (aOR 9.0, 95% CI: 3.1–26.1) were associated with increased in-hospital mortality.

**Conclusions:**

Certain patient characteristics and clinical features can help clinicians with early identification and triage of high-risk patients during subsequent waves of COVID-19.

## Introduction

The pandemic due to SARS-CoV-2, a newly described human coronavirus causing the disease known as COVID-19, continues to challenge the U.S. healthcare system. To date, there have been over 30,357,579 cases in the United States (US) and 138,255 cases in Rhode Island (RI), which has one of the highest infection rate per capita (12,910 cases per 100,000) in the country [1, 2]. In the early phase of the pandemic, many studies noted factors associated with severe outcomes [3–5]. Comorbidities like diabetes, obesity, and hypertension were observed to be prevalent in those with severe disease [6, 7]. We aimed to do an in-depth study of such predictors in the state of Rhode Island (RI). Such an understanding is crucial so that healthcare systems can manage the continued influx of patients with COVID-19 [8, 9] by appropriately triaging patients who present to the hospital.

We conducted a retrospective cohort study of persons with COVID-19 who were hospitalized in RI to identify (1) patient demographics and comorbidities associated with severe disease and death, and (2) presenting symptoms and vital signs that predicted progression to severe disease and death.

## Methods

### Study design and patient selection

We performed a retrospective cohort study of patients hospitalized with COVID-19 at the Lifespan academic hospitals affiliated with Brown University in Providence, RI. Patients of all ages who presented to the hospital with symptoms of COVID-19 and had a positive real time polymerase chain reaction (RT-PCR) result for SARS-CoV-2 were eligible for the study. Patients with asymptomatic infection or those who developed symptoms of COVID-19 after the first 48 hours of hospitalization were excluded.

**Fig 1:**
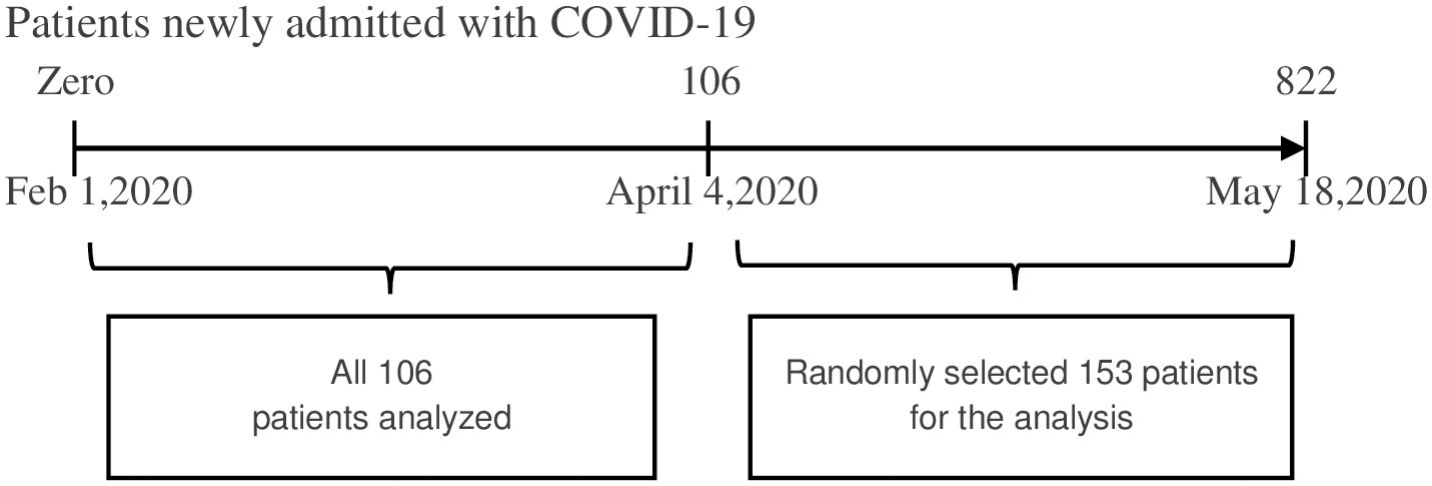
Participant selection: N = 259.

A list of medical record numbers (MRNs) was extracted from the integrated electronic medical record (EMR) for all patients with COVID-19 positive test results hospitalized between February 1, 2020 and May 18, 2020. There were 822 patients admitted during this time period. We included all 106 eligible patients who were hospitalized between February 17 to April 3 and a subset of patients hospitalized between April 4 and May 18, the peak of the pandemic surge in RI. For the latter group, we selected a random sample of 153 from the master patient list (Fig 1). In total, 259 patients, 31.5% of all patients admitted with COVID-19 during the time period, were included in this study. To assess representativeness of our cohort, we compared age, gender, and race among patients selected and not selected for the study. To ensure that we had a representative sample, we compared the weekly case fatality rates between the study sample and all COVID-19 patients admitted to the hospitals during the study period. We did not observe a statistical significance (Wilcoxon test P>0.1) [10].

The Lifespan Academic Medical Center institutional review board approved the study and waived informed consent for participation.

### Data collection

Demographic and laboratory data were extracted from the EMR. The following clinical data were collected by manual chart review and entered into a REDCap (Research Electronic Data Capture) database: baseline comorbidities, presenting symptoms and vital signs, microbiology results, imaging results, antimicrobial and other medical treatments (e.g. vasopressors), supplemental oxygen (O_2_), non-invasive and invasive forms of ventilation, intensive care unit (ICU) admission, renal replacement therapy, prior hospital visits, 15-day follow-up data (including telephone encounters and hospital re-admissions), co-infections, complications, and hospitalization outcome (death or discharge). Study team members were trained to perform chart reviews, and random charts were re-abstracted by the lead author to evaluate interrater agreement and calibrate data collection methods.

### Study outcomes

We defined severe COVID-19 as requiring high flow O_2_ (flow rate of more than 8L/min or use of high flow oxygen cannula), non-invasive ventilation (e.g. BIPAP), or invasive mechanical ventilation at any time point during the hospitalization. We evaluated four combinations of predictors and outcomes. Among all 259 patients, we examined differences in patient demographics and comorbidities in relation to (1) severe disease or (2) death at any time during hospitalization. To examine early clinical predictors of progression to severe disease and death, we excluded patients who met criteria for severe disease or died on the day of admission, and identified presenting symptoms, signs, laboratory results, and imaging findings during the first 24 hours that were associated with (3) developing severe disease or (4) death.

### Statistical analysis

Chi-square or Fisher’s exact tests for categorical variables and the Student’s t-test for continuous variables were used to compare demographics, comorbidities, and clinical data between groups. Variables that differed at a significance level of <0.30 were included in stepwise multivariable logistic regression analyses. Variables with a significance level of <0.35 were maintained in the model during stepwise selection. Estimated correlation matrix was used to check multicollinearity between clinically related variables. In multivariate regression, we excluded cases with ≤5 missing values for a given covariate and used the missing indicator method for variables with >5 missing observations. SAS 9.4 (SAS Institute, Cary, U.S.A.) and R 3.6.3 (R Computing, Vienna, Austria) were used for statistical analyses.

## Results

The 259 patients included in our cohort did not differ from the 563 patients not selected for the study with respect to age, gender, and race [Supplementary Materials]. Among the participants, median age was 62 years [interquartile range (IQR), 51–73]; 138 (53%) were male; 75 (29%) were Hispanic; and 53 (20.5%) Black [Table 1]. Sixty (23%) participants were admitted from a nursing facility; 52/259 (20%) had additional emergency room (ER) visits in the 15 days before hospitalization. The median length of stay for all patients was 8 days [IQR, 5–15]. ICU admission was required in 74/259 (28%); 42/259 (16%) patients required mechanical ventilation with a median time of 7 days [IQR, 3–12] on a ventilator.

**Table 1:** Demographics.

	n (%) or median [IQR]			
	All patients	Non-severe disease	Severe disease	p-*value*
	n = 259 (%)	n = 168(%)	n = 91(%)	
Age in years	62[51–73]	60[49–72]	65[57–74]	0.0162*
*Gender*				0.6930
Male	138(53.3)	88(52.4)	50(55.0)	
Female	121(46.7)	80(47.6)	41(45.1)	
*Ethnicity*				0.1246
Hispanic / Latino^a^	75 (29.0)	54 (32.1)	21 (23.1)	
Non-Hispanic/ Latino	184 (71.0)	114 (67.9)	70 (76.9)	
*Race*				
Black	53(20.5)	28(52.8)	25(47.2)	0.0396*
Non-Black	206(79.5)	140(68)	66(32.0)	
*Health care worker*				0.5899
Yes	15(5.8)	11(6.6)	4(4.4)	
No	178(68.7)	117(69.6)	61(67.0)	
Unknown	66(25.5)	40(23.8)	26(28.6)	
*Skilled nursing facility*				0.0059*
Yes	60(23.2)	30(17.9)	30(33.0)	
No	199(76.8)	138(82.1)	61(67.0)	

^a^5 with Black race also identified themselves as Hispanic/Latino.

*p-values of <0.05

Of the 259 participants, 91 (35%) had severe COVID-19, and 38 (15%) died at any time during hospitalization. Two hundred twenty-three participants did not have severe disease or die during the first 24 hours of admission. Among this group, 55 (24.7%) progressed to severe disease, and 24 (10.8%) died. Compared to the 168 participants who did not progress to severe disease, the 55 participants who progressed to severe disease had a higher incidence of arrhythmias (35% versus 5%; p<0.001) and death (40% vs. 1.2%, p<0.001). The differences in incidence of thromboembolic events (11% vs. 4%, p = 0.06) did not reach statistical significance and bleeding was rare (7% vs. 5%; p = 0.6).

Follow-up telehealth visits were completed in 124/221 discharged patients within a median time of 2 days [IQR, 2–4] post-discharge. At the time of follow-up visit, 84/124 (67%) still reported symptoms and 15/124 (12%) still required oxygen. Among all patients who survived, 16/221(7.2%) patients who were discharged required re-admission within 15 days.

We did not find any significant difference in distribution of black patients among remdesivir versus non remdesivir treatment groups (15.9 vs 17.4%, p-value 0.78) in our study.

### Baseline characteristics associated with severe COVID-19 at any time during hospitalization

Compared to patients with non-severe COVID-19, those with severe COVID-19 were older (65 [IQR 57–74] vs. 60 [IQR 49–72] years, p = 0.02) and more likely to be Black (47.2% vs. 32.0%, p = 0.04), admitted from a nursing facility (33.0% vs. 17.9%, p = 0.006), have diabetes (53.9% vs. 30.4%, p<0.001), or COPD (15.4% vs. 6.6%, p = 0.02) [Table 2]. No significant differences were noted with respect to gender, blood type, or current active immunosuppressive medication. In multivariate regression, only Black race (adjusted odds ratio [aOR] 2.0, 95% confidence interval [CI]: 1.1–3.9) and diabetes (aOR 2.2, 95% CI: 1.3–3.9) were found to be independent predictors of severe disease [Table 3].

**Table 2:** Medical comorbidities.

	n (%) or median [IQR]			
	All patients	Non-severe disease	Severe disease	p-value
	n = 259(%)	n = 168(%)	n = 91(%)	
Smoking history	93(35.9)	59(35.1)	34(37.4)	0.7194
Obesity^a^	114(44)	67(39.9)	47(51.7)	0.0686
Hypertension	164(63.3)	101(60.1)	63(69.2)	0.1463
Diabetes mellitus	100(38.6)	51(30.4)	49(53.9)	0.0002*
Pre-diabetes	38(14.7)	27(16.1)	11(12.1)	0.3871
Hyperlipidemia	134(51.7)	85(50.6)	49(53.9)	0.6172
Coronary artery disease	38(14.7)	27(16.1)	11(12.1)	0.3871
Cerebrovascular disease	22(8.5)	13(7.7)	9(9.9)	0.5532
Peripheral vascular disease	10(3.9)	5(3.0)	5(5.5)	0.3153
COPD^b^	25(9.7)	11(6.6)	14(15.4)	0.0215*
Asthma	30(11.6)	20(12.0)	10(11.0)	0.8260
Chronic kidney disease	45(17.4)	26(15.5)	19(20.9)	0.2733
Congestive heart failure	37(14.3)	20(11.9)	17(18.7)	0.1368
Chronic liver disease	16(6.2)	11(6.6)	5(5.5)	0.7368
Neurological diseases	53(20.5)	30(17.9)	23(25.3)	0.1578
Autoimmune disease	10(3.9)	8(4.8)	2(2.2)	0.3065
Organ transplant	10(3.9)	6(3.6)	4(4.4)	0.7424
HIV	5(1.9)	1(0.60)	4(4.4)	0.0792
Malignancy	29(11.2)	18(10.7)	11(12.1)	0.7379
Current immunosuppressive medication^c^	25(9.7)	13(7.7)	12(13.2)	0.1563
*Blood groups*^*d*^				0.4566
Blood group A	61(23.6)	39(23.2)	22(24.2)	
Blood group B	14(5.4)	6(3.6)	8(8.8)	
Blood group AB	6(2.3)	3(1.8)	3(3.3)	
Blood group O	73(28.2)	40(23.8)	33(36.3)	

^a^Obesity was defined as Body mass index (BMI)> 30kg/m^2^; BMI was missing in 11 patients

^b^COPD: Chronic Obstructive Pulmonary Disease

^c^Immunosuppressive medications included steroids, calcineurin inhibitors, mTOR inhibitors, antiproliferative agents, active chemotherapy, immunotherapy

^b^Blood group was missing in 105 patients

*p value<0.05

**Table 3:** Factors associated with severe disease.

	Adjusted Odds Ratio	95% Wald Confidence Limits		*p*-Value
Patient age at admission	1.011	0.993	1.030	0.2253
Black race vs. all others	2.016	1.052	3.864	0.0346*
Hospitalized from SNF^a^	1.825	0.932	3.575	0.0795
Obesity	1.444	0.820	2.544	0.2032
Diabetes mellitus	2.232	1.277	3.901	0.0048*
COPD	2.069	0.847	5.051	0.1104

Abbreviations:

^a^SNF, skilled nursing facility or rehabilitation

*p-value of <0.05, Multivariate stepwise regression for entire cohort (n=259)

### Patient characteristics associated with in-hospital mortality

Each one-year increase in patient age (aOR 1.04, 95% CI: 1.01–1.07) and admission from a skilled nursing facility (aOR 2.7, 95% CI 1.1–6.7) were associated with death during hospitalization. While many comorbidities were more common in the deceased group [Supplementary Materials], underlying hematological disorders (chronic anemia, coagulation disorders, hematological malignancies, and sickle cell disease) were the only comorbidity that predicted mortality in multivariate regression (aOR 3.4, 95% CI 1.1–10.0) [Table 4].

**Table 4:** Factors associated with in-hospital mortality.

	Adjusted Odds Ratio	95% Wald Confidence Limits		*p*-value
Patient age at admission	1.035	1.005	1.067	0.0242*
Hospitalized from SNF	2.742	1.130	6.654	0.0258*
Hypertension	1.859	0.662	5.220	0.2392
Diabetes mellitus	2.291	0.962	5.455	0.0611
Hyperlipidemia	0.520	0.203	1.333	0.1732
Peripheral Vascular Disease	3.051	0.674	13.823	0.1478
Asthma	0.121	0.013	1.135	0.0644
Hematological Disorders	3.373	1.134	10.036	0.0288*

*p-value of <0.05

Multivariate stepwise regression for entire cohort (n=259)

### Progression to severe disease after the first 24 hours of hospitalization

Among the 223 participants in this analysis, respiratory symptoms (94.6% vs. 83.3%, p = 0.04), tachypnea (85.5% vs. 64.9%, p = 0.003), and hypoxia (96.4% vs. 67.9%, p<0.0001) in the first 24 hours were associated with progressing to severe COVID-19 [Table 5]. For laboratory values, only estimated glomerular filtration rate (eGFR) <60 mL/min (48% versus 31%, p = 0.02), hypoalbuminemia (<3.5g/dl) (38% versus 17%, p = 0.01), and elevated D-dimer (>300ng/ml) (71% versus 44%, p = 0.02) were found to be significantly more common during the first 24 hours of admission among those who progressed to severe disease compared to those who did not [Table 6]. A higher proportion of patients who progressed to severe disease had abnormal findings on chest imaging, including bilateral disease on chest x-ray (44% versus 29%, p = 0.04) or chest CT (27% versus 15%, p = 0.03), and ground glass opacities on chest CT (35% versus 14%; p<0.001); a higher proportion of patients with non-severe disease had normal chest x-rays (23% versus 9%; p = 0.02) [Table 7]. In multivariate logistic regression, progression to severe disease was associated with respiratory symptoms (aOR 7.0, 95% CI: 1.4–34.1), hypoxia (aOR 19.9, 95% CI: 2.6–152.5), and hypotension (aOR 2.7, 95% CI) during first 24 hours [Table 8].

**Table 5:** Presenting symptoms and signs during the first 24 hours of admission.

	n (%) or median [IQR]			
	All patients	Non-severe disease	Severe disease	p-value
	n = 223(%)	n = 168(%)	n = 55(%)	
Days from symptom onset to presentation, median	5[8–3]	5[3–8]	7[2–9]	0.9277
*Subjective*				
Respiratory symptoms^a^	192(86.1)	140(83.3)	52(94.6)	0.0370*
GI symptoms^b^	118(52.9)	88(52.4)	30(54.6)	0.7801
Systemic symptoms^c^	178(79.8)	136(81.0)	42(76.4)	0.4617
Chest pain	52(23.3)	46(27.4)	6(10.9)	0.0015
*Objective*				
Fever^d^	133(59.6)	96(57.1)	37(67.3)	0.1838
Hypothermia^e^	21(9.4)	15(8.9)	6(10.9)	0.6625
Tachycardia^f^	118(52.9)	84(50.0)	34(61.8)	0.1275
Tachypnea^g^	156(70)	109(64.9)	47(85.5)	0.0039*
Hypoxia^h^	167(74.9)	114(67.9)	53(96.4)	< .0001*
Hypotension^i^	27(12.1)	14(8.3)	13(23.6)	0.0025*

^a^Symptoms of cough, shortness of breath, chest pain, sore throat, and congestion were grouped as respiratory;

^b^GI symptoms were nausea, vomiting, diarrhea, abdominal pain;

^c^systemic symptoms were fever, myalgias, rash, encephalopathy, dizziness.

Abbreviations: ^b^GI, gastrointestinal

^d^Fever was defined as the highest temp of >38C;

^e^hypothermia as the lowest temp of <36C;

^f^tachycardia was defined as having a heart rate of >100 beats per minute;

^g^tachypnea was defined as having a respiratory rate of >20 breaths per minute;

^h^hypoxia was defined as having an O2 saturation of <95% on room air;

^i^hypotension was having a systolic blood pressure of <90mm Hg.

*p-values of <0.05

**Table 6:** Laboratory values during the first 24 hours of admission.

	n (%) or median [IQR]			
	All patients	Non-severe disease	Severe disease	p-value
	n = 223	n = 168(%)	n = 55(%)	
Leukocytosis^a^	28(12.6)	20(12.1)	8(14.8)	0.4967
Leucopenia^b^	21(9.4)	14(8.4)	7(13.0)	
Normal WBC	171(76.7)	132(79.5)	39(72.2)	
Lymphopenia^c^	136(61)	98(59.4)	38(70.4)	0.2991
Thrombocytopenia^d^	48(21.5)	38(22.9)	10(18.5)	0.6189
ALT> 45 IU/L^e^	40(17.9)	29(21.2)	11(24.4)	0.6451
AST> 42 IU/L^e^	53(23.8)	35(25.6)	18(40.0)	0.0641
Elevated BUN^f^	57(25.6)	38(22.9)	19(35.2)	0.0733
Elevated creatinine^g^	54(24.2)	36(21.7)	18(33.3)	0.0841
eGFR<60^h^	76(34.1)	50(30.5)	26(48.2)	0.0182*
Hyponatremia^i^	97(43.5)	70(41.7)	27(49.0)	0.3350
Hypokalemia^j^	61(27.4)	50(30.1)	11(20.4)	0.3787
Hypocalcemia^k^	54(24.2)	38(22.9)	16(29.6)	0.4558
Elevated troponin^l^	32(14.3)	21(16.7)	11(25)	0.2234
Hypoalbuminemia^m^	40(17.9)	23(16.8)	17(37.8)	0.0116*
Elevated CRP^n^	112(50.2)	86(90.5)	26(100)	0.1028
Elevated D-dimer^o^	43(19.3)	28(43.8)	15(71.4)	0.0277*
Elevated Ferritin^p^	74(33.2)	51(82.3)	23(95.8)	0.1032
Elevated LDH^q^	73(32.7)	54(68.4)	19(86.4)	0.0951

Abbreviations: ^h^eGFR, estimated glomerular filtration rate; ^g^AKI/ CKD, acute kidney injury/ chronic kidney injury; ^f^BUN, blood urea nitrogen

^a^Leukocytosis is defined as WBC> 11x10exp9/L;

^b^leucopenia is defined as WBC< 3.5x10exp9/L;

^c^lymphopenia is defined as <1x10exp9/L;

^d^thrombocytopenia is defined as platelet count below 150x10exp9/L;

^f^elevated BUN defined as >24 mg/dl;

^g^Elevated creatinine defined as serum creatinine>1.27 mg/dl;

^i^hyponatremia is defined as serum sodium<135mEq/L;

^j^hypokalemia is defined as serum potassium < 3.5mEq/L;

^k^hypocalcemia is defined as serum calcium< 8.5 mEq/L;

^l^elevated troponin is defined as troponin of >0.06ng/ml;

^m^hypoalbuminemia is defined as serum albumin of <3.5 g/dl;

^n^elevated CRP (C-reactive protein) is defined as >10mg/L;

^o^elevated D-dimer is defined as >300ng/mL;

^p^elevated ferritin is defined as >120 ng/ml;

^q^elevated LDH defined as >220 IU/L

^o^D-dimer was missing in 138 patients; ^p^ferritin was missing in 137 patients; ^q^LDH was missing in 122 patients; ^n^CRP was missing in 102 patients; ^l^troponin was missing in 53 patients; ^e^ALT, ^e^AST, ^m^albumin was missing in 41 patients;

^h^eGFR was missing in 5 patients;

^c^lymphocyte count was missing in 4 patients; WBC count, ^d^platelet count, ^e^creatinine, ^f^BUN, ^k^calcium, and ^j^potassium was missing in 3 patients

*p-values of <0.05

**Table 7:** Radiology imaging results during the first 24 hours of admission.

	All patients	Non- severe disease	Severe disease	p-v*alue*
	n = 223(%)	n = 168(%)	n = 55(%)	
*CXR*^a^	218(97.8)			
Normal	44(19.7)	39(23.2)	5(9.1)	0.0223*
Unilateral abnormalities^b^	9(4)	6(3.6)	3(5.5)	0.5379
Bilateral abnormalities^c^	73(32.7)	49(29.2)	24(43.6)	0.0472*
Multifocal abnormalities^d^	58(26)	40(23.8)	18(32.7)	0.1907
Airspace disease^e^	145(65)	104(61.9)	41(74.6)	0.0880
Consolidation^e^	8(3.6)	6(3.6)	2(3.6)	0.9821
Ground glass^e^	9(4)	6(3.6)	3(5.4)	0.5379
Interstitial^e^	30(13.5)	24(14.3)	6(10.9)	0.5241
Nodular^e^	0(0)	0(0)	0(0)	NA^g^
Peripheral^e^	14(6.3)	10(6.0)	4(7.3)	0.7261
Pleural effusion^e^	9(4)	6(3.6)	3(5.5)	0.5379
*CT scan*^f^	70(31.4)			
Normal	6(2.7)	5(3.0)	1(1.8)	0.6450
Unilateral abnormalities^b^	0(0)	0(0.0)	0(0.0)	NA^g^
Bilateral abnormalities^c^	40(17.9)	25(14.9)	15(27.3)	0.0376*
Multifocal abnormalities^d^	25(11.2)	15(8.9)	10(18.2)	0.0590
Airspace disease^e^	28(12.6)	21(12.5)	7(12.7)	0.9648
Consolidation^e^	6(2.7)	3(1.8)	3(5.5)	0.1444
Ground glass^e^	43(19.3)	24(14.3)	19(34.6)	0.0009*
Interstitial^e^	1(0.4)	1(0.6)	0(0)	0.5663
Nodular^e^	5(2.2)	5(3.0)	0(0)	0.1957
Peripheral^e^	23(10.3)	17(10.1)	6(10.9)	0.8672
Pleural effusion^e^	8(3.6)	4(2.4)	4(7.3)	0.0904

^a^For chest x ray, laterality was not reported in 34 patients. Chest x ray was not done in 5 patients.

^b^Unilateral was the presence of abnormalities in one lung;

^c^bilateral was presence of abnormalities in both lungs;

^d^multifocal was abnormalities in multiple foci in same or both lungs. They are mutually exclusive.

^e^Descriptive variables, mutually exclusive

^f^CT scan was only done in 70 patients and laterality with or without multifocality was reported in all of them.

*p-value of <0.05

^g^Abbreviations: NA, non-applicable.

**Table 8:** Symptoms, signs, and findings associated with progression to severe disease. Multivariate stepwise regression for patients who did not have severe disease or die in the first day of hospitalization (n=223).^a^

Effect	Odds Ratio	95% Wald Confidence Limits		P-Values
Respiratory symptoms	7.008	1.442	34.070	0.0158*
Tachypnea	2.211	0.911	5.368	0.0796
Hypoxia	19.946	2.609	152.490	0.0039*
Hypotension	2.677	1.036	6.914	0.0420*
eGFR<60	1.974	0.937	4.162	0.0737
Elevated D-Dimer (Elevated vs Normal)	0.413	0.128	1.333	0.1392
Elevated D-Dimer (Missing vs Normal)	1.064	0.457	2.476	0.8861

^a^N = 223, Used in Regression = 218, Severe = 54, Non-Severe = 164; 5 observations were not used because of missing x-ray;

*p-value of <0.05

### Progression to death after the first 24 hours of hospitalization

On admission, chest pain, fever, elevated troponin, elevated BUN, elevated creatinine, and eGFR<60 were more common in patients who died during hospitalization, compared to those who were discharged [Supplementary Materials]. In the adjusted model, only the presence of tachypnea (aOR 8.7, 95% CI: 1.1–71.7) or hypotension (aOR 9.0, 95% CI: 3.1–26.1) during the first 24 hours were independently associated with in-hospital mortality [Table 9].

**Table 9:** Symptoms, signs, and findings associated with in-hospital mortality. Multivariate stepwise regression for patients who did not have severe disease or die in the first day of hospitalization (n=223).^a^

Effect	Adjusted Odds Ratio	95% Wald Confidence Limits		P-Values
Tachypnea	8.699	1.056	71.682	0.0444*
Hypoxia	3.353	0.409	27.468	0.2595
Hypotension	9.022	3.118	26.102	< .0001*
eGFR<60	2.559	0.949	6.902	0.0635

^a^N = 223, Used in Regression = 218, Severe = 54, Non-Severe = 164; 5 observations were not used because of missing x- ray;

*p-value of <0.05

## Discussion

In this study, we identified baseline patient characteristics that were associated with severe COVID-19 or death; and presenting signs and symptoms that were associated with progressing to severe COVID-19 or death after the first 24 hours of admission. These findings will help providers on the front lines of the pandemic triage patients and prioritize hospital resources.

Patients who developed severe disease were older, and age was an independent predictor of mortality, similar to findings from other studies [11–14]. We did not observe any significant effects of gender on outcomes. Multivariate regression revealed Black race to be an independent predictor of severe disease. Our findings are aligned with emerging literature on racial and socioeconomic disparities affecting COVID-19 outcomes [15–18]. Almost half of Black persons in our study developed severe disease, but Black race was not independently associated with increased mortality. Rather, advanced age or admission from a nursing home/rehabilitation center was associated with mortality, reflecting that the number and severity of comorbidities is an important driver behind risk of death during hospitalization. This finding underscores the importance of adjusting for age as well as comorbidities when interpreting the impact of race on mortality. Other studies from the U.S. also found that when adjusted for other covariates including age and comorbidities, Black race was not an independent predictor of death [15, 19]. While data from the United Kingdom (UK) [20] has also suggested increased severity of COVID-19 among those with Black race, provisional nationwide analysis from the UK points towards increased mortality even after adjusting for age and comorbidities [21]. Additional research is needed to determine the extent of racial disparities among persons who die from COVID-19.

Comorbidities like diabetes, hypertension, obesity, etc. have been associated with poor COVID-19 outcomes [13, 22–24]. In our study, comorbidities were also found to be associated with severe disease. Notably, while the prevalence of diabetes, obesity, and COPD was higher in those with severe disease, on adjusted regression analysis only diabetes was found to be an independent predictor of severe disease. Diabetes creates a hyperinflammatory state and impairs innate and cell-mediated immunity, which may predispose patients to the cytokine storm known to occur in severe COVID-19 [25, 26]. Furthermore, increased release of cytokines like interleukin-6 in patients with diabetes and COVID-19, in the face of possible blunted antiviral interferon responses and the delayed activation of Th1/Th17, may contribute to worse outcomes [27–29]. However, causality remains to be proven and severe manifestations could be reflective of other factors such as high viral burden, therefore preemptive use of immunosuppressive agents or IL-6 inhibitors remains controversial [30].

In addition to diabetes, the presence of hematological disorders was independently associated with mortality. Chronic anemia was the most common in this subgroup, followed by coagulation disorders, hematological malignancies, and sickle cell disease. This finding may be indicative of underlying chronic inflammation or baseline dysregulation of the coagulation/endothelial dysfunction interplay, which is another driver of severity in COVID-19 [26, 31, 32].

In our cohort, hypoxia in the first 24 hours was an independent predictor of progression to severe disease. Likewise, presence of tachypnea was also an early indicator of subsequent worsening. Hypotension predicted mortality in addition to clinical worsening. These findings underscore the importance of early frequent monitoring of vital signs, which could provide early clues of impending decompensation or death. Patients with these vital sign abnormalities merit close monitoring.

Higher prevalence of hypoalbuminemia in patients with severe COVID-19 likely reflects a catabolic state and critical illness [33]. We also noted eGFR<60 to be common in the severe group. The kidney damage could be due to direct cytopathic effects of the virus from ACE receptor mediated entry [34] or from hypotension. Elevations in D-dimer reflect thrombosis and abnormal coagulation cascade that is common in COVID-19 [35]. Chest x-rays were normal in many patients with non-severe disease. This could be due to lower sensitivity of chest x-ray earlier in the disease [36, 37]. Presence of bilateral infiltrates and ground glass opacities were also associated with disease progression. However, on multivariate regression analysis none of these lab makers or imaging findings independently predicted outcomes.

This study had limitations. First, we analyzed a subset of all patients admitted to our hospitals during the time period of interest, and thus may have introduced selection bias. However, selected patients did not differ from unselected patients with respect to demographics. Second, due to missing D-dimer, ferritin, LDH, and CRP in a subset of patients, we may not have captured associations between these laboratory values and outcomes. Third, imaging findings were compiled from radiology reports. Therefore, subjectivity in reporting style may affect whether or not our descriptive variables were used by the reporting radiologist. Finally, the evaluation of treatment was beyond the scope of this study, and the statistical models did not adjust for treatment. However, remdesivir and steroids, the treatments that have been shown to improve outcomes in patients with COVID-19 [38–43], were administered to a small percentage of patients with non-severe disease [Supplementary materials]. Therefore, treatment would not be expected to significantly alter our findings of factors associated with severe COVID-19, but we cannot confirm this hypothesis. Although racial disparities could affect treatment outcomes [44–46] we did not find any significant difference in distribution of black patients among remdesivir versus non remdesivir treatment groups in our study.

## Conclusions

In this cohort of hospitalized patients with COVID-19 in RI, Black race and diabetes were found to be independent predictors of severe disease. Older age, admission from nursing home or rehabilitation facilities, and presence of hematological disorders predicted mortality. Tachypnea, hypoxia, and hypotension in the first 24 hours predicted progression to severe disease or death later during the hospital stay. These findings can help clinicians with early identification and triage of high-risk patients in order to optimize the allocation of hospital resources.

## Supporting information

S1 Table Comparison of patients included and not included in the study. (DOCX)

S2 Table Demographics characteristics of patients who died vs. patients who were discharged. (DOCX)

S3 Table Medical comorbidities in patients who died vs. patients who were discharged. (DOCX)

S4 Table Presenting symptoms and signs during the first 24 hours of admission. (DOCX)

S5 Table Laboratory values during the first 24 hours of admission. (DOCX)

S6 Table Radiology imaging results during the first 24 hours of admission. (DOCX)

S7 Table Treatment received during hospitalization. (DOCX)
