# Supplementary material for: Predictors of severity and mortality among patients hospitalized with COVID-19 in Rhode Island
Source: PLoS One. 2021 Jun 18;16(6):e0252411. doi: 10.1371/journal.pone.0252411 (PMC8213072; doi:10.1371/journal.pone.0252411)
Supplement: S1 Table — (DOCX) [file pone.0252411.s001.docx]

S1 Table. Comparison of patients included and not included in the study.

|  | n (%) or median [IQR] | | | |
| --- | --- | --- | --- | --- |
|  | All patients  (n=822) | Included  (n=259) | Not Included  (n=563) | *p-value* |
| Age in years | 62 [49-74] | 62 [51-73] | 62 [47-75] | 0.1219 |
| *Gender* |  |  |  | 0.2940 |
| Male | 460 (56.0) | 138 (53.3) | 322 (57.2) |  |
| Female | 362 (44.0) | 121 (46.7) | 241 (42.8) |  |
| *Race* |  |  |  |  |
| Black | 142 (17.3) | 53 (20.5) | 89 (15.8) | 0.1010 |
| Non-Black | 680 (82.7) | 206 (79.5) | 474 (84.2) |  |
