# Supplementary material for: Predictors of severity and mortality among patients hospitalized with COVID-19 in Rhode Island
Source: PLoS One. 2021 Jun 18;16(6):e0252411. doi: 10.1371/journal.pone.0252411 (PMC8213072; doi:10.1371/journal.pone.0252411)
Supplement: S2 Table — (DOCX) [file pone.0252411.s002.docx]

S2 Table. Demographics Characteristics of Patients who Died vs. Patients who were Discharged.

|  | n (%) or median [IQR] | | | |
| --- | --- | --- | --- | --- |
|  | All patients  n=259 | Alive  n=221(%) | Deceased  n=38(%) | p-value |
| Age in years | 62[51-73] | 61 [49-71] | 72 [62-79] | <.0001* |
| *Gender* |  |  |  | 0.7910 |
| Male | 138(53.3) | 117 (84.8) | 21 (15.2) |  |
| Female | 121(46.7) | 104 (86.0) | 17 (14.0) |  |
| *Ethnicity* |  |  |  | 0.4378 |
| Hispanic / Latino^a^ | 75 (29.0) | 66 (88.0) | 9 (12.0) |  |
| Non-Hispanic/ Latino | 184(71.0) | 155 (84.2) | 29 (15.8) |  |
| *Race* |  |  |  |  |
| Black | 53(20.5) | 42 (79.3) | 11 (20.7) | 0.1605 |
| Non-Black | 206(79.5) | 179 (86.9) | 27 (13.1) |  |
| *Health care worker* |  |  |  | 0.2221 |
| Yes | 15(5.8) | 15 (100.0) | 0 (0.0) |  |
| No | 178(68.7) | 149 (83.7) | 29 (16.3) |  |
| Unknown | 66(25.5) | 57 (86.4) | 9 (13.6) |  |
| *Skilled nursing facility* |  |  |  | <.0001* |
| Yes | 60(23.2) | 40 (66.7) | 20 (33.3) |  |
| No | 199(76.8) | 181 (95.9) | 18 (9.1) |  |

^a^5 with Black race also identified themselves as Hispanic/Latino.

*p-values of <0.05
