# Supplementary material for: Predictors of severity and mortality among patients hospitalized with COVID-19 in Rhode Island
Source: PLoS One. 2021 Jun 18;16(6):e0252411. doi: 10.1371/journal.pone.0252411 (PMC8213072; doi:10.1371/journal.pone.0252411)
Supplement: S3 Table — (DOCX) [file pone.0252411.s003.docx]

S3 Table. Medical comorbidities in patients who died vs. patients who were discharged.

|  | n (%) or median [IQR] | | | |
| --- | --- | --- | --- | --- |
|  | All patients  n=259 | Alive  n=221(%) | Deceased  n=38(%) | p-value |
| Smoking history | 93(35.9) | 78 (35.3) | 15 (39.5) | 0.6198 |
| Obesity^a^ | 114(44) | 94 (42.5) | 20 (52.6) | 0.2467 |
| Hypertension | 164(63.3) | 132 (59.7) | 32 (84.2) | 0.0038* |
| Diabetes mellitus | 100(38.6) | 78 (35.3) | 22 (57.9) | 0.0082* |
| Pre-diabetes | 38(14.7) | 33 (14.9) | 5 (13.2) | 0.7752 |
| Hyperlipidemia | 134(51.7) | 109 (49.3) | 25 (65.8) | 0.0606 |
| Coronary artery disease | 38(14.7) | 32 (14.5) | 6 (15.8) | 0.8330 |
| Cerebrovascular disease | 22(8.5) | 20 (45.5) | 2 (22.2) | 0.1975 |
| Peripheral vascular disease | 10(3.9) | 6 (2.7) | 4 (10.5) | 0.0210* |
| COPD^b^ | 25(9.7) | 18 (8.1) | 7 (18.4) | 0.0475* |
| Asthma | 30(11.6) | 29 (13.1) | 1 (2.6) | 0.0620 |
| Chronic kidney disease | 45(17.4) | 34 (15.4) | 11 (28.9) | 0.0415* |
| Congestive heart failure | 37(14.3) | 27 (12.2) | 10 (26.3) | 0.0218* |
| Chronic liver disease | 16(6.2) | 13 (5.9) | 3 (7.9) | 0.6341 |
| Neurological diseases | 53(20.5) | 44 (19.9) | 9 (23.7) | 0.5942 |
| Autoimmune disease | 10(3.9) | 8 (3.6) | 2 (5.3) | 0.6272 |
| Organ transplant | 10(3.9) | 9 (4.1) | 1 (2.6) | 0.6702 |
| HIV | 5(1.9) | 4 (1.8) | 1 (2.6) | 0.3592 |
| Malignancy | 29(11.2) | 23 (10.4) | 6 (15.8) | 0.3311 |
| Hematological disorders | 25 | 16 (7.2) | 9 (23.7) | 0.0015* |
| *Blood groups^c^* |  |  |  |  |
| Blood group A | 61(23.6) | 52 | 9 | 0.5004 |
| Blood group B | 14(5.4) | 10 | 4 |  |
| Blood group AB | 6(2.3) | 4 | 2 |  |
| Blood group O | 73(28.2) | 59 | 14 |  |

^a^Obesity was defined as Body mass index (BMI)> 30kg/m^2^; BMI was missing in 11 patients

^b^COPD: Chronic Obstructive Pulmonary Disease

^c^Blood group was missing in 105 patients

*p value<0.05
