# Supplementary material for: Predictors of severity and mortality among patients hospitalized with COVID-19 in Rhode Island
Source: PLoS One. 2021 Jun 18;16(6):e0252411. doi: 10.1371/journal.pone.0252411 (PMC8213072; doi:10.1371/journal.pone.0252411)
Supplement: S4 Table — (DOCX) [file pone.0252411.s004.docx]

S4 Table. Presenting symptoms and signs during the first 24 hours of admission.

|  | n (%) or median [IQR] | | | |
| --- | --- | --- | --- | --- |
|  | All patients  n=223 | Alive  n=199(%) | Deceased  n=24(%) | p-value |
| *Subjective* |  |  |  |  |
| Respiratory symptoms^a^ | 192(86.1) | 171 (85.9) | 21 (87.5) | 0.8336 |
| GI symptoms^b^ | 118(52.9) | 104 (52.3) | 14 (58.3) | 0.5735 |
| Systemic symptoms^c^ | 178(79.8) | 157 (78.9) | 21 (87.5) | 0.1446 |
| Chest pain | 52(23.3) | 52 (100.0) | 0 (0.0) | 0.0016* |
| *Objective* |  |  |  |  |
| Fever^d^ | 133(59.6) | 114 (57.29) | 19 (79.8) | 0.0390* |
| Hypothermia^e^ | 21(9.4) | 18 (9.0) | 3 (12.5) | 0.5841 |
| Tachycardia^f^ | 118(52.9) | 103 (51.8) | 15 (62.5) | 0.3193 |
| Tachypnea^g^ | 156(70) | 133 (66.8) | 23 (95.8) | 0.0034* |
| Hypoxia^h^ | 167(74.9) | 145 (72.9) | 22 (91.7) | 0.0448* |
| Hypotension^i^ | 27(12.1) | 16 (8.0) | 11 (45.8) | <.0001* |

^a^Symptoms of cough, shortness of breath, chest pain, sore throat, and congestion were grouped as respiratory; ^b^GI symptoms were nausea, vomiting, diarrhea, abdominal pain; ^c^systemic symptoms were fever, myalgias, rash, encephalopathy, dizziness.

Abbreviations: ^b^GI, gastrointestinal

^d^Fever was defined as the highest temp of >38C; ^e^hypothermia as the lowest temp of <36C; ^f^tachycardia was defined as having a heart rate of >100 beats per minute; ^g^tachypnea was defined as having a respiratory rate of >20 breaths per minute; ^h^hypoxia was defined as having an O2 saturation of <95% on room air; ^i^hypotension was having a systolic blood pressure of <90mm Hg.

*p-values of <0.05
