# Supplementary material for: Predictors of severity and mortality among patients hospitalized with COVID-19 in Rhode Island
Source: PLoS One. 2021 Jun 18;16(6):e0252411. doi: 10.1371/journal.pone.0252411 (PMC8213072; doi:10.1371/journal.pone.0252411)
Supplement: S5 Table — (DOCX) [file pone.0252411.s005.docx]

S5 Table. Laboratory values during the first 24 hours of admission.

|  | n (%) or median [IQR] | | | |
| --- | --- | --- | --- | --- |
|  | All patients  n=223 | Alive  n=199(%) | Deceased  n=24(%) | p-value |
| Leukocytosis^a^ | 28(12.6) | 22 (78.6) | 6 (21.4) | 0.1263 |
| Leucopenia^b^ | 21(9.4) | 19 (90.5) | 2 (9.5) |  |
| Normal WBC | 171(76.7) | 156 (91.2) | 15 (8.8) |  |
| Lymphopenia^c^ | 136(61) | 120 (61.2) | 16 (69.6) | 0.2643 |
| Thrombocytopenia^d^ | 48(21.5) | 45 (22.8) | 3 (13.0) | 0.3307 |
| ALT> 45 IU/L^e^ | 40(17.9) | 37 (22.8) | 3 (15.0) | 0.4244 |
| AST> 42 IU/L^e^ | 53(23.8) | 45 (27.8) | 8 (40.0) | 0.2563 |
| Elevated BUN^f^ | 57(25.6) | 44 (22.3) | 13 (56.5) | 0.0004* |
| Elevated creatinine | 54(24.2) | 44 (22.3) | 10 (43.5) | 0.0258* |
| eGFR<60^h^ | 76(34.1) | 62 (31.8) | 14 (60.9) | 0.0057* |
| Hypokalemia^j^ | 61(27.4) | 57 (28.9) | 4 (17.4) | 0.4936 |
| Hypocalcemia^k^ | 54(24.2) | 47 (23.9) | 7 (30.4) | 0.7111 |
| Elevated troponin^l^ | 32(14.3) | 23 (15.4) | 9 (42.9) | 0.0026* |
| Hypoalbuminemia^m^ | 40(17.9) | 32 (19.6) | 8 (40.0) | 0.1148 |
| Elevated CRP^n^ | 112(50.2) | 104 (92.0) | 8 (100.0) | 0.4067 |
| Elevated D-dimer^o^ | 43(19.3) | 37 (48.1) | 6 (75.0) | 0.1091 |
| Elevated Ferritin^p^ | 74(33.2) | 67 (84.8) | 7 (100.0) | 0.2663 |
| Elevated LDH^q^ | 73(32.7) | 65 (69.9) | 8 (100.0) | 0.0665 |

Abbreviations: ^h^eGFR, estimated glomerular filtration rate; ^g^AKI/ CKD, acute kidney injury/ chronic kidney injury; ^f^BUN, blood urea nitrogen

^a^Leukocytosis is defined as WBC> 11x10exp9/L; ^b^leucopenia is defined as WBC< 3.5x10exp9/L; ^c^lymphopenia is defined as <1x10exp9/L; ^d^thrombocytopenia is defined as platelet count below 150x10exp9/L; ^f^elevated BUN defined as >24 mg/dl; ^g^Elevated creatinine defined as serum creatinine>1.27 mg/dl; ^j^hypokalemia is defined as serum potassium < 3.5mEq/L; ^k^hypocalcemia is defined as serum calcium< 8.5 mEq/L; ^l^elevated troponin is defined as troponin of >0.06ng/ml; ^m^hypoalbuminemia is defined as serum albumin of <3.5 g/dl; ^n^elevated CRP (C-reactive protein) is defined as >10mg/L; ^o^elevated D-dimer is defined as >300ng/mL; ^p^elevated ferritin is defined as >120 ng/ml; ^q^elevated LDH defined as >220 IU/L

^o^D-dimer was missing in 138 patients; ^p^ferritin was missing in 137 patients; ^q^LDH was missing in 122 patients; ^n^CRP was missing in 102 patients; ^l^troponin was missing in 53 patients; ^e^ALT, ^e^AST, ^m^albumin was missing in 41 patients; ^h^eGFR was missing in 5 patients; ^c^lymphocyte count was missing in 4 patients; WBC count, ^d^platelet count, ^e^creatinine, ^f^BUN, ^k^calcium, and ^j^potassium was missing in 3 patients

*p-values of <0.05
