# Supplementary material for: Predictors of severity and mortality among patients hospitalized with COVID-19 in Rhode Island
Source: PLoS One. 2021 Jun 18;16(6):e0252411. doi: 10.1371/journal.pone.0252411 (PMC8213072; doi:10.1371/journal.pone.0252411)
Supplement: S6 Table — (DOCX) [file pone.0252411.s006.docx]

S6 Table. Radiology imaging results during the first 24 hours of admission.

|  | All patients  n=223(%) | Alive  n=199(%) | Deceased  n=24(%) | p-*value* |
| --- | --- | --- | --- | --- |
| *CXR^a^* | 218(97.8) | 195(98) | 23(95.8) | 0.5002 |
| Normal | 44(19.7) | 42 (21.1) | 2 (8.3) | 0.0784 |
| Unilateral abnormalities^b^ | 9(4) | 7 (3.5) | 2 (8.3) | 0.1906 |
| Bilateral abnormalities^c^ | 73(32.7) | 64 (32.2) | 9 (37.5) | 0.5985 |
| Multifocal abnormalities^d^ | 58(26) | 53 (26.6) | 5 (20.8) | 0.5406 |
| Airspace disease^e^ | 145(65) | 129 (64.8) | 16 (66.7) | 0.8581 |
| Consolidation^e^ | 8(3.6) | 7 (3.5) | 1 (4.2) | 0.3960 |
| Ground glass^e^ | 9(4) | 7 (3.5) | 2 (8.3) | 0.1906 |
| Interstitial^e^ | 30(13.5) | 27 (13.6) | 3 (12.5) | 0.2497 |
| Nodular^e^ | 0(0) | 0(0) | 0(0) | NA^g^ |
| Peripheral^e^ | 14(6.3) | 13 (6.5) | 1 (4.2) | 0.3485 |
| Pleural effusion^e^ | 9(4) | 7 (3.5) | 2 (8.3) | 0.1906 |
|  |  |  |  |  |
| *CT scan^f^* | 70(31.4) | 61 (31.0) | 9 (39.1) | 0.4262 |
| Normal | 6(2.7) | 6 (3.0) | 0 (0.0) | 0.5008 |
| Unilateral abnormalities^b^ | 0(0) | 0(0) | 0(0) | NA^g^ |
| Bilateral abnormalities^c^ | 40(17.9) | 36 (18.1) | 4 (16.7) | 0.2222 |
| Multifocal abnormalities^d^ | 25(11.2) | 21 (10.6) | 4 (16.7) | 0.1618 |
| Airspace disease^e^ | 28(12.6) | 25 (89.3) | 3 (12.5) | 0.2533 |
| Consolidation^e^ | 6(2.7) | 5 (2.5) | 1 (4.2) | 0.3817 |
| Ground glass^e^ | 43(19.3) | 37 (18.6) | 6 (25.0) | 0.1523 |
| Interstitial^e^ | 1(0.4) | 1 (0.5) | 0 (0.0) | 0.8924 |
| Nodular^e^ | 5(2.2) | 5 (2.5) | 0 (0.0) | 0.5628 |
| Peripheral^e^ | 23(10.3) | 22 (11.1) | 1 (4.1) | 0.1961 |
| Pleural effusion^e^ | 8(3.6) | 5 (2.5) | 3 (12.5) | 0.0375* |

^a^For chest x ray, laterality was not reported in 34 patients. Chest x ray was not done in 5 patients.

^b^Unilateral was the presence of abnormalities in one lung; ^c^bilateral was presence of abnormalities in both lungs; ^d^multifocal was abnormalities in multiple foci in same or both lungs. They are mutually exclusive.

^e^Descriptive variables, mutually exclusive

^f^CT scan was only done in 70 patients and laterality with or without multifocality was reported in all of them.

*p-value of <0.05

^g^Abbreviations: NA, non-applicable
