# Supplementary material for: Predictors of severity and mortality among patients hospitalized with COVID-19 in Rhode Island
Source: PLoS One. 2021 Jun 18;16(6):e0252411. doi: 10.1371/journal.pone.0252411 (PMC8213072; doi:10.1371/journal.pone.0252411)
Supplement: S7 Table — (DOCX) [file pone.0252411.s007.docx]

S7 Table. Treatment received during hospitalization.

|  | n (%) or median [IQR] | | | |
| --- | --- | --- | --- | --- |
|  | All patients  n=223 | Non severe  n=168(%) | Severe group  n=55(%) | p-value |
| Antibiotics^a^ | 153 | 103(61.3) | 50(90.9) | <0.0001* |
| Azithromycin | 117 | 77(45.8) | 40(72.7) | 0.0005* |
| Time to onset of azithromycin, median days [IQR] | 1[1-2] | 1[1-2] | 1[1-3] | 0.7725 |
| Azithromycin duration days | 3[2-5] | 3[1-5] | 3[2-5] | 0.2948 |
| Remdesivir | 63 | 39(23.2) | 24(44.4) | 0.0026* |
| Remdesivir duration, median[IQR] | 7[5-10] | 6[4-9] | 9[6-10] | 0.0243* |
| Time to onset of Remdesvir, median [IQR] | 3[2-3] | 2[2-3] | 3[2-4] | 0.6747 |
| Hydroxychloroquine | 38 | 24(14.5) | 14(25.5) | 0.0610 |
| Hydroxychloroquine duration, median [IQR] | 5[3-6] | 5[3-6] | 4[2-5] | 0.1176 |
| Convalescent plasma | 12 | 5(3.0) | 7(12.7) | 0.0054* |
| Time to onset of plasma, median days [IQR] | 2[2-3] | 2[1-2] | 3[2-3] | 0.6908 |
| Steroids | 45 | 23(13.7) | 22(40) | <0.0001* |
| Median duration of steroids | 5[2-8] | 4[2-8] | 5[3-8] | 0.3266 |
| Time to onset of steroids | 2[1-4] | 2[1-2] | 2[1-8] | 0.0494* |
| Anticoagulation | 197 | 142(84.5) | 55(100) | <0.0019* |
| Need for pressor | 21 | 1(0.6) | 20(37.0) | <0.0001* |
| Need for renal replacement | 4 | 0(0) | 4(7.3) | 0.0004* |

^a^Antibiotics other than azithromycin; *p-value of <0.05
